# Characterization of anthocyanins, phenolics, and flavonoids in a global carrot collection through application of chemometrics and FT-NIR spectroscopy

**DOI:** 10.1016/j.fochx.2025.102807

**Published:** 2025-07-18

**Authors:** Romit Seth, Chelsey Fiecke, Guoying Ma, Penelope Perkins-Veazie, Pablo Cavagnaro, Mario G. Ferruzzi, Massimo Iorizzo

**Affiliations:** a Plants for Human Health Institute, North Carolina State University, Kannapolis, North Carolina, United States; b Arkansas Children's Nutrition Center and Department of Pediatrics, University of Arkansas for Medical Sciences, Little Rock, AR, 72202, United States; c Department of Horticultural Science, North Carolina State University, Raleigh, North Carolina, United States; d National Scientific and Technical Research Council (CONICET), National Agricultural Technology Institute (INTA) E.E.A. Mendoza, Mendoza 5507, Argentina

**Keywords:** Purple carrot, Acylated anthocyanins, Chemometrics, High-throughput phenotyping, Near-infrared spectroscopy, Phenolic acids

## Abstract

Purple carrot is an important source of anthocyanins as a natural food dye, yet research advancements in selecting purple cultivars with improved anthocyanin extractable yield and stability remain limited. This study used a large set of purple carrot accessions to characterize anthocyanins, phenolics, and flavonoids, their relationship to tissue-specific accumulation, extraction efficiency, color density, and color stability. A Fourier-transform near-infrared (FT-NIR) spectroscopy method was also developed to evaluate these metabolites. Fully purple roots showed the highest anthocyanin content and extraction efficiency. Non-acylated anthocyanin Cy3XG was identified as a limiting step in the biosynthesis of stable acylated anthocyanins*.* The sinapoyl-containing glycoside Cy3XSGG was the primary contributor to color density. Ten FT-NIR prediction models were developed to evaluate anthocyanins, chlorogenic, and caffeic acids. These findings support the selection of purple carrot with stable and uniform anthocyanin production and perform quality control in the natural colorant industry.

## Highlights

• Fully purple roots accumulate the highest amount of anthocyanin and phenolic acids.
• Fully purple roots have the highest anthocyanin extraction efficiency.
• Cy3XG is limiting step to accumulate higher amount of stable acylated anthocyanins.
• Acylated anthocyanin Cy3XSGG contributes the most to color density.
• Ten FT-NIR models predicted anthocyanins and phenolics accurately.

## 1 Introduction

In recent years, purple carrots have received much interest as a natural source of anthocyanin (ACNs) for applications in the food colorant industry (Iorizzo et al., 2020). Previous studies have demonstrated that carrot primarily accumulate five types of cyanidins, including three acylated forms—cyanidin 3-O-xylosyl(coumaroylglucosyl)galactoside (Cy3XCGG), cyanidin 3-O-xylosyl(feruloylglucosyl)galactoside (Cy3XFGG), and cyanidin 3-O-xylosyl(sinapoylglucosyl)galactoside; and two non-acylated forms—cyanidin 3-O-xylosylgalactoside (Cy3XG) and cyanidin 3-O-xylosylglucosylgalactoside (Cy3XGG) (Algarra et al., 2024; Cavagnaro & Iorizzo, 2019; Pérez et al., 2023). ACN extracted from purple carrot exhibit low or no off flavors, and some genetic stocks contain high ACN content (up to 18 mg/100 g fresh weight) (Montilla et al., 2011). Purple carrot can contain 29 %–88 % more ACN than anthocyanin-rich barley, corn, radish, potato, sweet potato, and tomato cultivars (Butelli et al., 2008; Ginting et al., 2020; Giusti et al., 1998; Jian et al., 2019; Kim et al., 2007; Lago et al., 2015). Further, carrot ACN offers a wide range of color opportunities, ranging from strawberry pink at low pH to purple and blue shades at higher pH (Buchweitz, 2024; Kirca et al., 2007; Kırca et al., 2006). Depending on the genotype, purple carrot can accumulate a high percentage of acylated anthocyanin (AcA), which are the major ACN pigments used in the food color industry due to their increased stability at higher pH and longer shelf-life (Giusti & Wrolstad, 2003; Kammerer et al., 2004). Multiple studies have shown that carrot AcA were more stable than non-acylated anthocyanins (NAA) at multiple storage temperatures and pH ranges evaluated (Bąkowska-Barczak, 2005; Kamiloglu et al., 2015a; Kamiloglu et al., 2015b; Kamiloglu et al., 2018; Perez et al., 2022; Turker et al., 2004; Türkyılmaz & Özkan, 2012). For example, in a study comparing the stability of ACNs in a soft drink stored at different temperature, ACNs extracted and purified from purple carrot degraded less compared to ACNs extracted from Andean blackberries and acai (Zozio et al., 2011). This color stability was attributed to the AcA present in purple carrot (Perez et al., 2022). Besides ACNs, carrot contain phenolic acids (PHENs) and/or flavonoids (FLVs), which can stabilize the anthocyanin quinonoid base through intramolecular and intermolecular co-pigmentation and metal chelation (Houghton et al., 2021). The presence of potential co-pigments, offers potential advantage for enhancing the stability of both acylated and non-acylated cyanidins (Gras et al., 2018; Zeng et al., 2024).

Despite these advantages, carrot ACN extracts are often derived from carrots that are not fully purple, or cultivars not specifically selected for pigment yield and stability and large amount of AcA. This variability limits the colorant industry's ability to optimize ACN yield and color stability, thereby contributing to high cost and inconsistent quality of natural ACN based colorants. Previous studies evaluated ACN composition in carrot and highlighted a large phenotypic variation in terms of ACN content and their tissue-specific accumulation in the root (Blando et al., 2021; Pérez et al., 2023; Valerga et al., 2023). Although highly useful, the expensive analytical methods required to characterize the carrot germplasm has limited the number of purple samples analyzed (*N* = 29). Moreover, ACN content in relation to tissue-specific accumulation or extraction efficiency has not been explored, and the diversity of potential co-pigments remain uncharacterized.

To address these gaps, this study utilized a large set of purple carrot to characterize ACN, PHENs and FLVs composition and assess their relationship to tissue-specific accumulation and extraction efficiency. This material represents the largest and most diverse set of purple carrot germplasm evaluated to date. Additionally, a high-throughput Fourier-Transform Near Infrared (FT-NIR) method was developed to evaluate ACN and PHENs, offering a cost-effective high-throughput method to screen purple germplasm in breeding programs and for quality control in the natural colorant industry. The outcomes of this study provide new insights into the ACN biosynthetic flux in carrot, providing guidance for the selection of purple material to better meet the needs of the colorant industry for increased stability, uniformity, and yield of ACNs production.

## 2 Materials & methods

### 2.1 Plant material and samples preparation

**Fig. 1:**
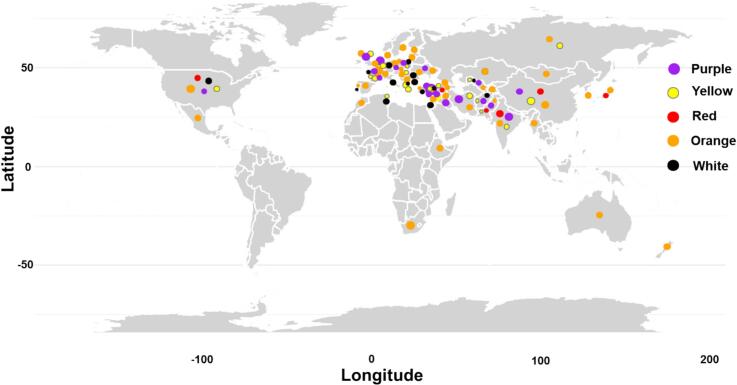
Distribution map of 461 carrot samples derived from 400 accessions and commercial cultivars.

In total, 400 accessions and commercial cultivars were grown at the NC Horticulture Research Station, Clinton (NC) (35.0009° N, 78.3374° W) during the winter of 2021 and spring of 2022. Seeds of carrot accessions were obtained from the USDA germplasm repository and the UK germplasm repository, while commercial cultivars were purchased from seed companies **(Table S1)**. Accessions and commercial cultivars were selected to maximize the representation of the purple phenotype and to represent the five population groups identified by Coe et al. (2023) that span the global carrot genetic/phenotypic diversity from 56 countries (Fig. 1).

Following harvest, 3–10 roots from each plot/accession were stored at 4 °C and subsequently processed to evaluate root color and analyze anthocyanins, phenolics and flavonoids. Roots samples were washed in running water, dried, cut into horizontal and vertical sections and photographed using a Canon EOS Rebel T5. In each picture, an x-rite color checker card was used (https://www.xrite.com). Multiple roots were processed and kept as separate samples for accessions or commercial cultivars with different color phenotypes (e.g., root with purple skin, non-purple root) within the same plot. After photography, each root was cut into small pieces (cubes), held at −80 °C, and then freeze dried for 10 days in a VirTis Lyotroll freeze dryer (SP Scientific). Freeze dried samples were homogenized using 2010 Geno/Grinder® (SPEX SamplePrep) for 2 min at 1500 rpm × 2 cycles and stored at −80 °C until further use. A total of 461 samples from 400 accessions were used for visual color phenotyping and targeted metabolites and NIR analysis.

### 2.2 Root color phenotyping

Fresh roots were phenotyped by scoring a binary trait for the presence (“1”) or absence (“0”) of root coloration (purple) in each root tissue, consisting of 1) periderm/skin; 2) cortex; 3) phloem; and 4) xylem. Scores were further verified using photographic images. For the image analysis, the 3D surface plot of color spectra of each phenotype was generated using matplotlib and OpenCV libraries (Bradski, 2000; Hunter, 2007). The binary data obtained from this evaluation was used to categorize all the samples in different groups of purple phenotypes. For instance, samples with only purple skin were grouped in the group “:1000”.

### 2.3 NIRS analysis

Near-infrared spectroscopy (NIRS) spectral data were collected using a Bruker Optics MPA FT-NIR Spectrometer (Billerica, MA, USA). Aliquots of 3.5 g of homogenized freeze-dried powder from each sample, prepared in triplicates, were transferred into glass vials (117,723, Bruker Optics, MA, USA). The final spectra were obtained as the mean of 64 scans with a resolution of 16 cm^−1^, which ranged from 3594 cm^−1^ to 12,489 cm^−1^ at room temperature (22–23 °C) and 35–40 % relative humidity.

### 2.4 UPLC-MS/MS analysis

Samples used for NIRS were utilized for quantitative estimation of phenolics, anthocyanins and flavonoids using ultra-performance liquid chromatography–tandem mass spectrometry (UPLC-MS/MS) analysis.

#### 2.4.1 Phenolic/anthocyanin extraction and analysis

##### 2.4.1.1 Chemicals and standards

LC-MS grade solvents (water, methanol, acetonitrile, isopropanol) were purchased from Fisher Scientific (Waltham, MA, USA), formic acid was purchased from Sigma-Aldrich (St. Louis, MO, USA), glacial acetic acid from Alfa Aesar (Haverhill, MA, USA), and sodium metabisulfite and citric acid from Fisher Scientific (Hampton, NH, USA). Authentic reference standards for phenolic compounds and anthocyanins were purchased from Sigma-Aldrich, BenchChem (Austin, TX, USA), or BioCrick (Chengdu, China) **(**Table. 1**).**

**Table 1:** Standards of metabolites used and their abbreviations.

Metabolite Category	Metabolites Standards used	Abbreviations
**Phenolic acid (PHEN)**	ferulic acid	**FA**
	4-hydroxybenzoic acid	**4HBA**
	p-coumaric acid	**p-cou**
	caffeic acid	**CA**
	sinapic acid	**SA**
	3-p-coumaroylquinic acid	**3pcou**
	neochlorogenic acid (5-O-caffeoylquinic acid)	**5CQA**
	chlorogenic acid (3-O-caffeoylquinic acid)	**3CQA**
	cryptochlorogenic acid (4-O-caffeoylquinic acid)	**4CQA**
	3-O-feruloylquinic acid	**3FQA**
	5-O-feruloylquinic acid	**5FQA**

**Non-acylated anthocyanins (NAA)**	pelargonidin 3-O-glucoside	**pel3glu**
	cyanidin 3-O-glucoside	**cy3glu**
	peonidin 3-O-glucoside	**peo3glu**
	delphinidin 3-O-glucoside	**del3glu**
	cyanidin 3-O-xylosylgalactoside	**Cy3XG**
	cyanidin 3-O-xylosylglucosylgalactoside	**Cy3XGG**

**Acylated anthocyanins (AcA)**	cyanidin 3-O-xylosyl(coumaroylglucosyl)galactoside	**Cy3XCGG**
	cyanidin 3-O-xylosyl(feruloylglucosyl)galactoside	**Cy3XFGG**
	cyanidin 3-O-xylosyl(sinapoylglucosyl)galactoside	**Cy3XSGG**

**Flavonoids (FLV)**	quercetin 3-O-galactoside	**q3gal**
	quercetin 3-O-glucoside	**q3glu**
	kaempferol 3-O-rutinoside	**k3rut**

##### 2.4.1.2 Semi-automated high throughput extraction of anthocyanins and other phenolic compounds

A standard method for extraction of anthocyanins and other phenolic compounds, as described by Mengist et al. (2020) and Mohamedshah et al. (2020), was modified to accommodate higher throughput with an automated fluid-handling robot (Tecan EVO 150, Tecan; Mannedorf, Switzerland). An aliquot (∼100 mg) of carrot powder was weighed in a 5 mL polyethylene vial (SPEX SamplePrep, Metuchen, NJ, USA) containing 5–10 steel beads (2.38 mm, Qiagen, Germantown, MD, USA). Vials were placed into Tecan sample racks (16 tubes/rack) and loaded into defined Tecan columns. After the addition of 1 mL of methanol:water:formic acid (*v*/v, 78:20: 2), samples were homogenized using a 2010 Geno/Grinder® (SPEX SamplePrep) for 2 min at 1750 rpm. The homogenate was centrifuged (Eppendorf 5910 Ri, Barkhausenweg, Germany) for 4 min at 4000 rpm. Vials and 16 × 100 mm glass culture tubes were placed into Tecan racks for transfer of supernatant. The residue was extracted two more times using 2 % formic acid in 98: 2 methanol and water. The combined supernatants were dried under N2 and resolubilized in 5 mL of 0.1 % formic acid in water. Resolubilized samples (750 μL) were loaded onto AcroPrepTM 96-well filter plates (1 mL, 0.45 μm Supor membrane, Cytiva, Marlborough, MA, USA). All samples were spiked with 100 μL of ethyl gallate (1 mg/mL) for the determination of extraction recovery.

A solid phase extraction (SPE) procedure was performed to remove sugars that may cause a decrease in sensitivity of the MS system. Prior to SPE, filtered extracts were diluted (1:10) in 1 % formic acid in water. The SPE plate (Phenomenex, StrataX 33 μm, 10 mg/well) was preconditioned with 1 % formic acid in methanol, followed by 1 % formic acid in water. Diluted samples (500 μL) were transferred to the SPE plate and spiked with 20 μL taxifolin (0.2 mM) as internal standard to determine SPE recovery. Samples were drained by using a positive pressure manifold. The SPE plate was washed with 1 mL of 1 % formic acid in water followed by 1 mL of 0.1 % formic acid in water. Elution of targeted phenolic compounds was completed with 300 μL of 0.1 % formic acid in methanol, then diluted (1:1) with 0.1 % formic acid in water. An aliquot (160 μL) of diluted sample was transferred to a 96-round well, polypropylene 350 μL collection plate (Waters, Milford, MA, USA). Twenty μL of taxifolin (0.2 mM) aliquot was added as a volume control to adjust for any variation in volume between wells following SPE. The SPE recovery was in the range of 71–97 % with an average recovery of 79 ± 7 %. After accounting for SPE recovery, total extraction recovery was found to range from 64 to 105 % with an average recovery of 93 ± 11 %.

##### 2.4.1.3 Analysis of anthocyanins and phenolic compounds by UPLC-MS/MS

Anthocyanins and phenolic compounds in carrot powders were analyzed by UPLC-MS/MS using a method adapted from Gras et al. (2016) and Mohamedshah et al. (2020). Samples were injected into a Waters ACQUITY Premier UPLC system equipped with an ACQUITY UPLC BEH C18 column (1.7 μm, 2.1 × 50 mm). Target compounds were separated using a flow rate of 0.5 mL min^−1^ using a gradient elution profile based on a binary phase of 2 % formic acid in water (solvent A) and 0.1 % formic acid in acetonitrile (solvent B). Separation was achieved at 40 °C using the following gradient: initially 100 % A, 0–0.5 min 100–94 % A, 0.5–2.0 min 94–91 %, 2.0–3.0 min 91–87 %, 3.0–4.5 min 87–65 %, 4.5–5.0 65–100 % A, 5.0–6.5 min 100 % A. Following separation, individual anthocyanins and phenolic compounds were detected using a Waters Xevo TQ-S micro instrument by means of multiple reaction monitoring (MRM) in both negative and positive ion mode. Compounds detected under negative mode electrospray ionization (ES-) used the following conditions: desolvation temperature 600 °C, desolvation gas flow 800 L h^−1^, capillary voltage −2.5 kV, cone voltage 32 V, and collision energy 3 V. Compounds detected under positive mode electrospray ionization (ES+) used the same conditions, except capillary voltage was 3 kV. The MS source parameters, including cone voltage and collision energy, were optimized by directly infusing individual standards of target compounds. A total of 29 compounds were selected and targeted for analysis **(Table S2).** Authentic standards of each compound were used to determine concentrations of compounds within samples and were used to quantify these compounds via calibration curves developed from individual MRM responses **(Table S3)**. Final concentrations were adjusted by each individual sample extraction recovery as determined by internal standard responses. Validation parameters, including limit of detection (LOD), limit of quantitation (LOQ), and coefficient of variation (CV), are reported in **Table S3 and S4**.

### 2.5 NIRS model development and model evaluation

NIRS models were used to predict total anthocyanins (ACN), total acylated anthocyanins (AcA), total phenolic acids (PHEN), 3CQA, CA, and the individual anthocyanin pigments, Cy3XSGG, Cy3XFGG, Cy3XCGG, Cy3XG, Cy3XGG (full names of abbreviated phenolic and anthocyanin compounds are presented in Table 1, S3 and S4) using the software OPUS – Quant2 (www.brucker.com). The NIRS spectral data were imported into the software along with the UPLC quantification data, which acted as the reference/true value. The raw spectral data were pre-processed using standard normal variate (SNV) to remove the background noise from the spectra. The partial least square regression (PLSR) method was used to build a calibration models by correlating spectral data with UPLC-MS/MS data. To evaluate model robustness, a 5-fold cross-validation approach (k **= 5)** was applied using the caret package in R (Kuhn, 2015). The model accuracy and robustness were evaluated by the coefficient of determination (R^2^) and root mean square error of cross-validation (RMSECV) (Sadergaski et al., 2023). The performance of the predicted models was also evaluated by calculating the residual prediction deviation (RPD), wherein a higher value of RPD indicates higher accuracy of models (Larson et al., 2023). In addition to the cross-validation method, prediction models for ACN were also developed and validated using the external validation method. For this, spectra data from a subset of 50 purple samples (in triplicates) were used to develop a calibration curve, and 100 purple samples (in triplicates) were used for external validation. This analysis was performed using the software OPUS – Quant2 (www.brucker.com).

### 2.6 Extraction efficiency and color stability in purple carrots

#### 2.6.1 Preparation of fresh purple carrot and color extracts

Fresh carrot samples were prepared from individual roots of 40 purple carrot cultivars and accessions by pureeing using a food processor (Hamilton Beach) **(Table S5)**. A composite sample of each type was used and then separated into three technical replicates (120 total). Pureed carrots were freeze-dried for 16 h at 29 °C (Harvest Right LLC, Salt Lake City, Utah). A bench-scale model of commercial color extraction was developed using a solution of 2 % acetic acid containing 3000 ppm SO_2_ (pH 2.8). Three technical replicates of fresh purple carrot puree from each cultivar (20 g each) were steeped in the acetic acid solution for 3 h in amber glass bottles using a New Brunswick I2400 incubator oscillating at 180 rpm at 25 °C. A 20 mL aliquot of each steeped extract was filtered using a cellulose acetate filter tube (0.45 μm) centrifuged at 3000 x*g* for 5 min at 4 °C. Filtered extracts were transferred to a Petri dish and freeze-dried for 16 h as described above. Solids content was determined in freeze-dried aliquots of fresh purple carrot puree and steeped purple carrot color extracts. Color extracts were prepared by reconstituting 100 mg of each dried extract in 0.1 M citric acid (pH 2.0) normalized to 10 % solids. Reconstituted extracts were centrifuged at 10000 x*g* for 10 min at 4 °C, filtered using a PTFE syringe filter (0.45 μm, 15 mm; Macherey-Nagel), then stored in amber HPLC vials at 4 °C until analysis. The ACN and PHENs were analyzed by UPLC-MS/MS as mentioned in section 2.4.1.2 and 2.4.1.3.

#### 2.6.2 Determination of extraction efficiency and color stability for anthocyanins and other phenolic compounds

Concentrations of individual anthocyanins and phenolic compounds were determined in freeze dried purple carrot purees and color extracts on a dry weight basis. Concentrations were normalized to solids content of the puree (μg/ 100 g dw). In color extracts, concentrations were then adjusted for solids extraction recovery (%). Extraction efficiency was calculated as: (concentration in steeped color extract / concentration in freeze dried carrot puree) x 100 %. The color density (CD) and hue (H) of color extracts was determined according to the method of Mazza et al. (1999). Briefly, absorbance was measured at 420, 520, and 700 nm using a microplate reader (BMG Labtech, POLARstar Omega) for 200 μL aliquots of each color extract, in triplicate. The CD and hue were calculated using the equations below.

$$\mathrm{CD}=\left(A_{420}-A_{700}\right)+\left(A_{520}-A_{700}\right)$$

$$H=\left(A_{520}-A_{700}\right)/\left(A_{420}-A_{700}\right)$$

CD was used to measure color intensity, which reflects both oxidized and non-oxidized anthocyanin content. Hue is an index of browning and oxidation (lower value indicates greater degree of browning).

### 2.7 Data analysis

The S.H.E analysis (S: Species richness, H: Shannon-weiner diversity index and E: Shannon-weiner evenness) was used to assess the visual binary phenotypic diversity across all carrot accessions and derived samples (461) in this study (Wakefield, 2003). Multivariate analyses were used to evaluate the relationship between metabolite content and binary data (Usman et al., 2021). This included principal component analysis (PCA) and partial least square discriminant analysis (PLS-DA) implemented using the prcomp, chemometrics.R and plsr functions in R (Wehrens & Mevik, 2007). Pearson's correlation analysis was used to assess the association between the metabolite content, binary phenotypes, CD, hue, and extraction efficiency. All experiments were conducted in triplicate and multifactor statistical significance was assessed using Tukey's HSD (Abdi & Williams, 2010).

## 3 Results and discussion

### 3.1 Root color phenotyping

**Fig. 2:**
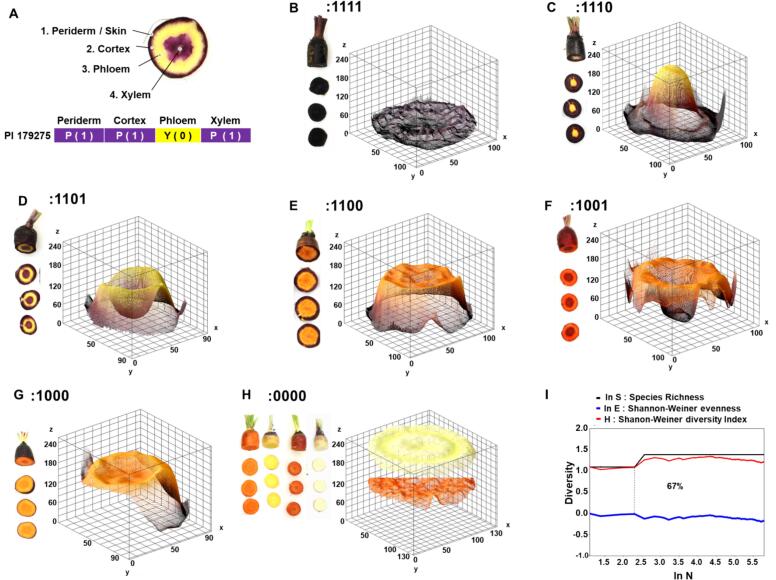
Root color phenotypic diversity. **(A)** Example of tissue-specific phenotyping based on presence/absence of purple coloration in four tissues of root tissues: Periderm (skin) → Cortex → Phloem → xylem. **(B—H)** Representation of the seven carrot phenotypic categories identified based on presence or absence of purple coloration in the four root tissues, accompanied by respective 3D surface plot generated using matplotlib and openCV (Bradski, 2000; Hunter, 2007). The seven category include: complete purple roots (:1111) (**B**); purple root with non-purple xylem (:1110) **(C)**; non-purple phloem (:1101) **(D)**; purple periderm and cortex (:1100) **(E)**; purple periderm and xylem (:1001) **(F)**; only purple periderm (:1000) **(G)**; and non-purple roots including orange, yellow, red and white (:0000) **(H)**. **(I)** The S.H.E analysis across 461 carrot samples. (For interpretation of the references to color in this figure legend, the reader is referred to the web version of this article.)

Of the 461 samples (400 accessions) from 56 countries, 296 were non-purple (18 red; 182 orange; 64 yellow; 32 white), and 165 were purple in at least one of the root tissues **(**Fig. 1**; Table S1)**. Seven phenotype combinations were identified based on the presence/absence of purple coloration in the four tissues (periderm or skin, cortex, phloem and xylem) **(**Fig. 2A**)**. This included fully-purple roots (:1111), roots with all purple tissues except for xylem (:1110), roots with all purple tissues except for phloem (:1101), roots with purple periderm and cortex but non-purple phloem and xylem (:1100), roots with purple periderm and xylem but non-purple cortex and phloem (:1001), roots with purple periderm and the rest of the tissues non-purple (:1000), and completely non-purple roots (:0000) **(**Fig. 2B-H**).**

The origins of purple accessions/cultivars were distributed across 16 countries with maximum representation from Turkey (78), India (42) Iran (23) and Afghanistan (17) **(**Fig. 1**)**. Analysis of the phenotypic data using the Shannon-Weiner evenness indices estimated 67 % phenotypic diversity, highlighting a substantial level of pigmentation variability among samples **(**Fig. 2I**)**. The positive values of log S and H indicate a phenotypically well-distributed and diverse root sample essential for capturing the full spectrum of purple pigmentation, particularly for identifying anthocyanin-rich accessions/genotypes. Additionally, evenness values (E) close to zero suggest a balanced distribution of the seven binary phenotypes, probably minimizing potential bias in machine learning models for spectral analysis (Bal et al., 2024). This balance enhances the predictive power of NIR-based models, allowing for accurate differentiation of pigmentation variations across diverse carrot accessions/cultivars. In addition, given that this material represents the most diverse set of purple carrots ever evaluated in a single study, it provides a unique opportunity to assess the relationship between visual purple phenotypes and anthocyanin, phenolic acids, and flavonoid metabolites content and composition.

### 3.2 Chemometric analysis of Total anthocyanins, phenolic acids and flavonoids

**Fig. 3:**
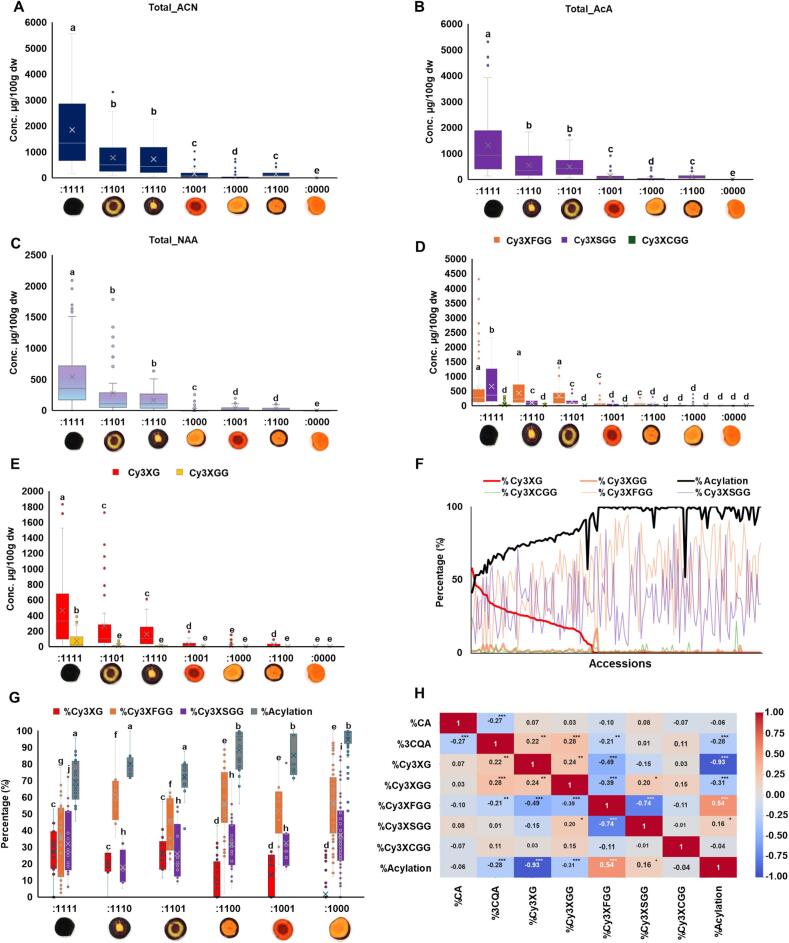
Anthocyanin diversity. **(A-E)** Distribution, median and mean concentrations (μg/100 g dw) of **(A)** Total ACNs, **(B)** Total AcA, **(C)** Total NAA, **(D)** acylated forms Cy3XFGG, Cy3XSGG and Cy3XCGG and **(E)** non-acylated Cy3XG and Cy3XGG across the seven purple root phenotypic categories. **(F)** Graph representing a trend of degree of acylation (% Acylation) with % Cy3XG, %Cy3XFGG, %Cy3XSGG and %Cy3XCGG. **(G)** Boxplot representing variation in %Acylation, % Cy3XG, %Cy3XFGG, %Cy3XSGG and %Cy3XCGG across 6 purple phenotypes. **(H)** Pearson's correlation plot showing correlation among %CA, %3CQA, %Acylation, % Cy3XG, %Cy3XFGG, %Cy3XSGG and %Cy3XCGG. The values in the plot represent the Pearson's correlation coefficient (r), and significance is represented as *p < 0.05, **p < 0.01 and ***p < 0.001. Statistical significance across phenotypes in boxplots were assessed using Tukey's HSD test, where different letters indicate significant differences at *p* < 0.05. (For interpretation of the references to color in this figure legend, the reader is referred to the web version of this article.)

Anthocyanins and phenolic acids were the most abundant target metabolites evaluated in this study. The anthocyanins detected across all samples consist of mono-acylated (AcA) and non-acylated (NAA) derivatives of cyanidins. Pelargonidin, peonidin and delphinidin derivatives were detected in trace amounts in a few samples and below the detection threshold for most of the samples. On average, the total ACN content ranged from 0.5 μg/100 g dw in samples with non-purple root (:0000) to 1799.7 μg/100 g dw in samples with complete purple phenotype (:1111) **(**Fig. 3A**)**. In the fully purple phenotype, total ACN content ranged from 225.7 to 4527.1 μg/100 g dw. The fully purple samples ‘Super Black’ and ‘Deep Purple’ had the highest amount of total ACN followed by ‘Black Nebula’ and ‘Pusa Asita’. ‘Black Nebula’ and ‘Pusa Asita’ were reported to have the highest concentration of total ACN by Pérez et al. (2023) in a study with 27 purple samples; ‘Deep Purple’ and ‘Super Black’ were not part of that study. As ‘Black Nebula’ and ‘Pusa Asita’ contain very high levels of total ACN, these cultivars represent a potential target value for ACN pigments for the food industry. The low diversity of anthocyanidins detected in the large set of purple carrots in this study confirms previous studies indicating that cyanidin represents the predominant anthocyanidins in carrot as reported earlier by Algarra et al. (2014), Blando et al. (2021), and Montilla et al. (2011). As suggested by Iorizzo et al. (2020), low diversity in anthocyanidins detected in carrot may be due to the lack of *flavonoid 3′,2′-hydroxylase* and *anthocyanidin reductase,* two genes that are responsible for the structural differentiation of anthocyanidins in plants (Ma et al., 2021; Yildiz et al., 2013). However, it is important to note that in a few studies, extremely low amounts of peonidin (Peonidin 3-xylosylglucosylgalactoside and its Ferulic acid derivative) and pelargonidin (Ferulic acid derivative of pelargonidin 3-xylosylglucosylgalactoside) have been identified in carrot ACN extracts (Mizgier et al., 2016). However, in this study, those ACN were not detected, either because standards for those ACN were not included in the analysis or because they were present at a concentration that is below the detection threshold.

The ACN composition play an important role in food colorant applications. The ACN detected here included three AcA, sinapoyl derivative cyanidin 3-O-xylosyl(sinapoylglucosyl)galactoside (Cy3XSGG), feruloyl derivative (Cy3XFGG), and p-coumaroyl derivative (Cy3XCGG); and two NAA, cyanidin 3-O-xylosylgalactoside (Cy3XG) and cyanidin 3-O-xylosylglucosylgalactoside (Cy3XGG). Acylated ACN from carrots are more stable than non-acylated ACN in food colorant applications (Ebrahimi Tirtashi et al., 2019; Perez et al., 2022; Zeng et al., 2024). For the vast majority of the purple carrot samples analyzed, AcAs predominated over NAAs, in agreement with previous reports (Algarra et al., 2014; Montilla et al., 2011; Pérez et al., 2023) **(**Fig. 3B-C**)**. Total AcA represent the most abundant fraction of ACN in fully purple roots (average1266.5 μg/100 g dw) **(**Fig. 3B**).** The fully purple root cultivars ‘Super Black’, ‘Deep Purple’, ‘Black Nebula’, ‘Pusa Asita’, ‘Black Knight’, ‘9913.11’, ‘F3 population of F.S. x ((2566B xTrksh) x 9304))’ and ‘PI 652336’ had the highest amounts of AcA (1150.4 to 4328.1 μg/100 g dw). Among AcA, Cy3XSGG and Cy3XFGG were the most abundant pigments, while the p-coumaroyl derivative (Cy3XCGG) was the least abundant, confirming previous studies (Cavagnaro et al., 2014; Curaba et al., 2020; E. Gläßgen et al., 1992; Koga et al., 2024; Pérez et al., 2023). The average content of Cy3XSGG and Cy3XFGG ranged from 0.2 and 0.3 μg/100 g dw, respectively, in samples with a non-purple root (:0000), to 658.4 μg/100 g dw and 620.1 μg/100 g dw, respectively, in samples with a fully purple phenotype (:1111) **(**Fig. 3D**)**. The cultivars ‘Deep Purple’ and ‘Super Black’ and accessions ‘PI 204575’ and ‘PI 652336’ showed higher accumulation of Cy3XFGG, while ‘Black Nebula’, ‘Black Knight’, ‘Pusa Asita’ and ‘15’b122–1’ exhibited a more than threefold higher content of Cy3XSGG. Comparatively, Cy3XCGG was low, averaging 32.9 μg/100 g dw in the fully purple phenotype (:1111) **(**Fig. 3D**)**. The percentage of Cy3XFGG ranged from 2.3 % in ‘Black Nebula’ to 77.8 % in ‘Deep Purple’, while Cy3XSGG ranged from 6.2 % in ‘Deep Purple’ to 72.6 % in ‘Pusa Asita’. NAAs Cy3XG and Cy3XGG averaged 465.4 μg/100 g dw and 74.5 μg/100 g dw, respectively, and were detected at significantly higher concentrations in roots with a fully purple phenotype (:1111) **(**Fig. 3E**)**.

The degree of acylation across samples ranged from 40 % to near 100 % (Fig. 3F)**.** The fully purple roots had a significantly lower degree of acylation, with an average value of 69.5 %, compared to roots with only purple periderm, which exhibited the highest degree of acylation (94.6 %) **(**Fig. 3G**)**. Among the fully purple cultivars, ‘Super Black’, ‘Deep Purple’ were an exception, with >85 % and 95 % AcA, respectively. The carrot accessions ‘PI 478861’, ‘PI 652189’, ‘PI 200876’, ‘Dragon’ and ‘PI 17968’ with only purple periderm were found to have 100 % of acylation despite lower content of total ACN. Interestingly, the percentage of NAA Cy3XG was strongly negatively correlated (*r* = −0.9, *p* < 0.001) with the degree of acylation **(**Fig. 3F-H**)**. This inverse relationship suggests that the conversion from Cy3XG to Cy3XGG may be the main limiting factor in production and accumulation of very high fractions of AcA. Among the carrot samples with highest total ACN, only ‘Super Black’ and ‘Pusa Asita’ (95.5 % and 87.6 % AcA, respectively) had no detectable amount of Cy3XG, indicating that these genotypes may be used as a source to select or produce very high amount of AcA.

The percentage of Cy3XFGG and Cy3XSGG were inversely correlated (*r* = −0.8, p < 0.001; Fig. 3H**)**, which is likely due to the interplay between the competitive use of the same substrate (Cy3XGG) for the acylation reaction using feruloyl or sinapoyl donors and the presence/absence of specific genes controlling the conversion into these two different forms of AcA. For instance, a Serine Carboxypeptidase-Like (*DcSCPL1*) can convert Cy3XGG into both Cy3XSGG and Cy3XFGG, while a specific gene like UDP-glucose: sinapic acid glucosyltransferase (*DcUSAGT*) has been reported to carry the glycosylation of sinapic acid to produce 1-O-sinapoylglucose that serve as an acyl donor in acylation of anthocyanins to generate Cy3XSGG (Chen et al., 2016; Curaba et al., 2020; Koga et al., 2024).

**Fig. 4:**
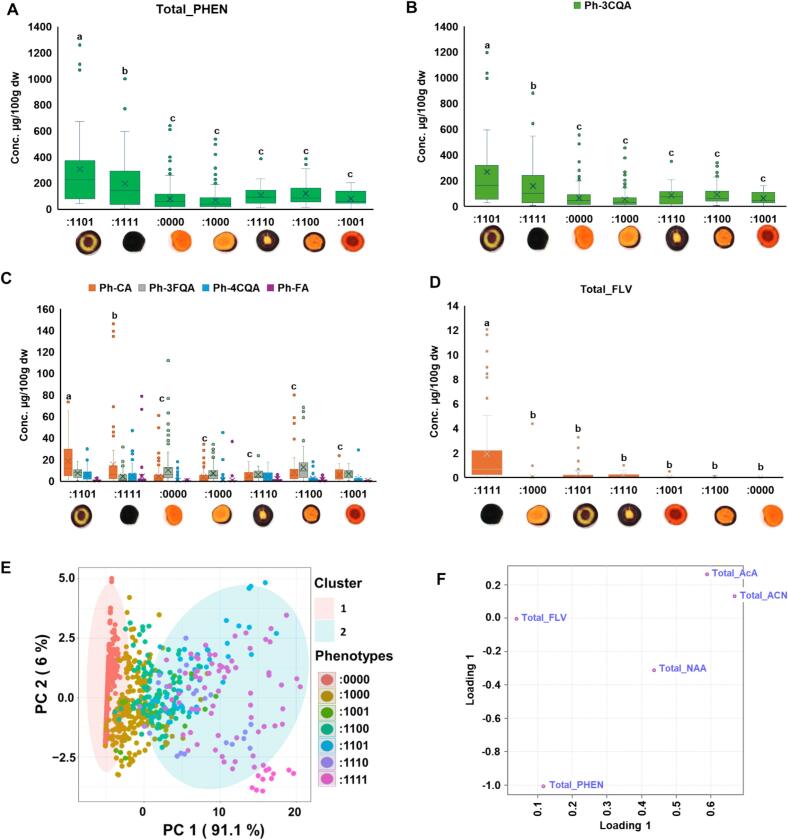
Phenolics, flavonoids and anthocyanin diversity and clustering. **(A-E)** Distribution, median and mean concentrations (μg/100 g dw) of **(A)** Total PHEN, **(B)** 3CQA, **(C)** other PHENs and **(D)** total FLVs across the seven phenotypes. **(E)** PCA analysis using root color phenotypes, anthocyanins, phenolics and flavonoids data. **(F)** PC1 loading scores. Statistical significance across phenotypes in boxplots were assessed using Tukey's HSD test, and different letters indicate significant differences at *p* < 0.05.

Phenolics and flavonoids compounds can function as co-pigments of anthocyanins and increase the stability of the latter (Gras et al., 2018; Zeng et al., 2024). The total PHEN content ranged from 69 μg/100 g dw in carrot roots with only purple skin/periderm (:1000) to 308.2 μg/100 g dw in purple roots with non-purple phloem (:1101) **(**Fig. 4A**)**. Among the PHENs, 3-O-caffeoylquinic acid (3CQA), also known as chlorogenic acid was the most abundant metabolite, followed by caffeic acid (CA), while 3-O-feruloylquinic acid (3FQA) was present in relatively lower amount **(**Fig. 4B-C**)**. Other PHEN such as 4-O-caffeoylquinic acid (4CQA), ferulic acid (FA), 4-hydroxybenzoic acid (4HBA), p-coumaric acid (p-cou), 3-p-coumaroylquinic acid (3pcou), sinapic acid (SA), 5-O-caffeoylquinic acid (5CQA), and 5-O-feruloylquinic acid (5FQA), were detected in trace amount and were excluded for downstream analysis. Earlier studies have also reported higher accumulation of 3CQA followed by CA, and relatively lower or trace accumulation of other PHENs in purple carrot roots (Blando et al., 2021; Scarano et al., 2018).

The correlation between PHENs and ACNs was positive but moderate (*r* = 0.45, p < 0.01), which increased slightly when considering only purple carrots (*r* = 0.49, p < 0.001) **(Fig. S1)**. On average, PHENs were most abundant in samples with purple roots with non-purple phloem (:1101) followed by fully purple roots (:1111), and these two phenotypes had higher PHENs than the rest of the root color phenotypes. The maximum average amount of 3CQA (268.8 μg/100 g dw) and CA (18.4 μg/100 g dw) was detected in purple root with non-purple phloem (:1101), while 3FQA had a maximum average content of only 12.9 μg/100 g dw in purple root with non-purple phloem and xylem (:1100) **(**Fig. 4B-C**)**. The purple root accessions ‘PI 652335’, ‘PI 204575’, and individuals of the ‘F3 population of F.S. x ((2566B xTrksh) x 9304))’ had the highest amount of 3CQA. These three accessions also have yellow phloem. In a previous study, Scarano et al. (2018) also found highest 3CQA levels in purple carrot roots with non-purple (yellow) phloem and xylem. Despite this observation, some cultivars with fully purple root and highest amount of ACN like ‘Super Black’, exhibited very high PHENs (731 μg/100 g dw).

Total flavonoid (FLV) content was relatively low in all samples **(**Fig. 4D**)**. The main FLV was quercetin 3-O-galactoside (q3gal) and quercetin 3-O-glucoside (q3glu), detected at a maximum concentration of 9.0 μg/100 g dw and 0.4 μg/100 g dw, respectively, while kaempferol 3-O-rutinoside (k3rut) was not detected. Given that total FLVs were detected at very low levels and primarily in fully purple samples, this group of metabolites were not used for downstream analysis.

Principal component analysis (PCA) using all visual phenotypes and metabolites indicated that PC1 and PC2 explain over 96 % of the variance, with PC1 explaining most of the variance (91.1 %). Along the PC1, the fully purple phenotype (:1111) and non-purple phenotypes (:0000) grouped into two opposite clusters, while all the other purple phenotypes grouped into an intermediate cluster **(**Fig. 4E**)**. As expected, the PCA loading plot indicated that total ACN and AcA are major factors separating purple and non-purple phenotypes **(**Fig. 4F**)**. In contrast, no clustering was observed along the PC2, and the main variant explaining PC2 was total PHENs.

### 3.3 Prediction of anthocyanins and phenolic acids with NIRs models

**Fig. 5:**
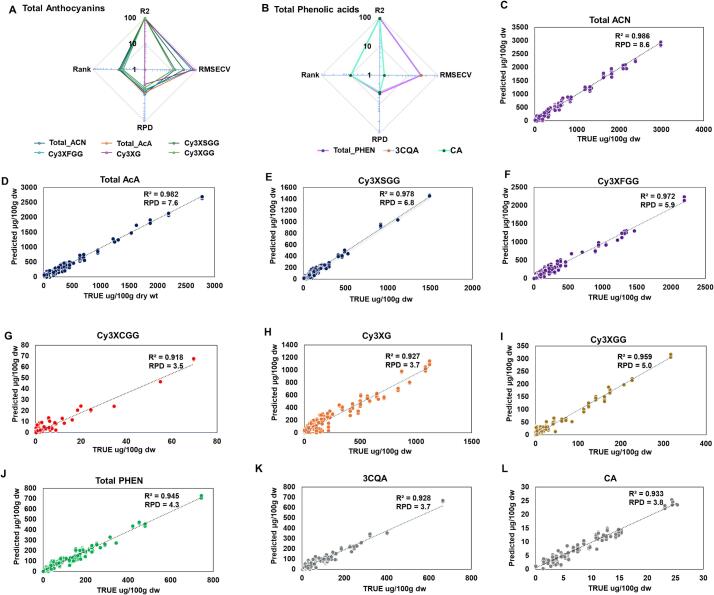
NIR prediction models. (A) Assessment of predictive partial least squared models of **(A)** total anthocyanins and **(B)** total phenolic acids in diverse purple carrot accessions using spectral scans obtained between 12,489 cm^−1^ to 3594 cm^−1^ at room temperature (22–23 °C) and 35–40 % relative humidity. Correlation between predicted vs. true model for **(C)** Total anthocyanins, **(D)** Total acylated anthocyanins, (**E)** Cy3XSGG, **(F)** Cy3XFGG **(G)** Cy3XCGG; non acylated anthocyanins (**H)** Cy3XG & (**I)** Cy3XGG; **(J)** Total Phenolic acids, (**K)** 3-O-caffeoylquinic acid: 3CQA and **(L)** Caffeic acid. R^2^: coefficient of determination in cross validation; RMSECV: root mean square error of cross-validation; RPD: residual prediction deviation from the Rank. (For interpretation of the references to color in this figure legend, the reader is referred to the web version of this article.)

Only carrots with purple root (*N* = 160) were used for NIR analysis. For each purple root, three independent samples were used to collect NIR spectra. These spectral data were used to build and evaluate Fourier Transform Near Infrared (FT-NIR) spectroscopy model for quantifying ACNs and PHENs. FT-NIR offers a non-destructive, cost effective, and efficient alternative to quantify these metabolites, streamlining the analysis process without compromising accuracy (Park et al., 2024). To assess the accuracy of the developed NIR prediction models, the coefficient of determination (R^2^) between the predicted and measured contents (true) of ACNs and PHENs was employed, along with the Residual Prediction Deviation (RPD) as quality checks (Arslan et al., 2023; Jiang & Zhu, 2013; Larson et al., 2023). Overall, ten optimized PLSR models for total ACN, total AcA, Cy3XFGG, Cy3XSGG, Cy3XCGG, Cy3XG, Cy3XGG, total PHENs, 3CQA and CA were developed using UPLC-MS/MS as the reference method. The cross-validation results showed high prediction accuracies, with R^2^ ranging from 0.92 to 0.98 and RPD from 3.5 to 8.6 **(**Table 2**;** Fig. 5A-B**)**. A model with an RPD value greater than 6 is considered to have excellent predictive ability, while an RPD of more than 2 represents good predictive ability (Morellos et al., 2016). The spectral data were normalized to remove the random noise occurring from factors like fluctuations in temperature and humidity or inherent characteristics of the samples (Park et al., 2024). The wavelength range of 8925.6–12,489.7 cm^−1^ and 4482.1–7159 cm^−1^, was correlated with both total ACN and AcA contents, consistently with previous findings reporting that similar spectral ranges corresponded to anthocyanins in bilberry(Gardana et al., 2018). The predicted models exhibited a slight positive bias (total ACN: 0.4 & AcA: 0.4) with Root Mean Square Error of Cross-Validation (RMSECV) values of 84.1 and 66.4 for total ACN and total AcA, respectively, showing slight overestimation and variability in predicted values (Fig. 5A-D**)**. However, given the high R^2^ and RPD values, the overall accuracy of the PLSR models for predicting AcA and NAA remains strong, highlighting the effectiveness of the spectral analysis approach. The estimates of total ACN and total AcA suggest strong concordance between the predicted and true values with statistical significance at 95 % confidence interval **(Table S6)**.

**Table 2:** Performance statistics of FT-NIR predicted models of anthocyanins and phenolics.

ACNs & PHENs	R^2^	RPD	RMSECV
Total_ACN	0.98	8.6	84.1
Total_AcA	0.98	7.6	66.4
Total_PHEN	0.94	4.3	27.2
Cy3XSGG	0.98	6.8	32.4
Cy3XFGG	0.97	5.9	65.2
Cy3XCGG	0.92	3.5	3.33
Cy3XG	0.96	3.7	79.4
Cy3XGG	0.93	5.0	12.9
3CQA	0.93	3.7	26.8
CA	0.93	3.8	1.45

Among the AcA, PLSR model for Cy3XSGG, Cy3XFGG and Cy3XCGG displayed high predictive accuracy with R^2^ values of 0.98, 0.97, and 0.92, respectively **(Table: 2;** Fig. 5E-G**)**. The post-normalization spectra data were significantly correlated with Cy3XSGG content in the region between 9820.5 and 10,715.5 cm^−1^ and 4482.1–6264.1 cm^−1^. Similarly, Cy3XFGG prediction used the first derivative and vector normalization showing correlation between 8925.6 and 12,489.7 cm^−1^, 7151.3–8046.2 cm^−1^, and 5369.2–6264.1 cm^−1^. Cy3XCGG was associated with spectral ranges of 11,594.8–12,489.7 cm^−1^, 7151.3–10,715.4 cm^−1^, and 4482.1–5377 cm^−1^. The high RPD values (Cy3XFGG: 5.9; Cy3XSGG: 6.8; Cy3XCGG: 3.5) of predicted models further validated the robustness of these models, indicating strong concordance between predicted and true values at a 95 % confidence interval **(Table S7)**.

Among the NAA, the PLSR model of Cy3XG and Cy3XGG also demonstrated strong predictive accuracy with R^2^ > 0.9 and RPD > 3 **(**Table 2**;** Fig. 5H- I**)**. The wavelength range 11,594.8–12,489.7 cm^−1^ and 8038.4–10,715.4 cm^−1^ showed significant correlation with both Cy3XG and Cy3XGG content. The PLSR model for Cy3XGG (R^2^: 0.96; RMSECV: 12.9; RPD: 5.0) displayed higher predictive accuracy compared to Cy3XG (R^2^: 0.93; RMSECV: 79.4; RPD: 3.7) **(**Table 2**, S6)**. Overall, the results indicated optimum performance of the model with strong concordance between the predicted and measured (true) value, with statistical significance at 95 % confidence interval **(Table S7).**

For ACN, in addition to the cross-validation method, prediction models were also developed and validated using the external validation method. The PLSR models the models continued to demonstrate strong predictive accuracy. For total anthocyanin content, the model achieved R^2^ₚ = 0.95 and RPDₚ = 4.39 **(Fig. S2A)**. Similar results were obtained for the for individual anthocyanins, Cy3XFGG (R^2^ₚ = 0.96, RPDₚ = 4.91), Cy3XSGG (R^2^ₚ = 0.95, RPDₚ = 4.54), Cy3XCGG (R^2^ₚ = 0.93, RPDₚ = 3.85), Cy3XG (R^2^ₚ = 0.93, RPDₚ = 3.75) and Cy3XGG (R^2^ₚ = 0.94, RPDₚ = 4.16) **(Fig. S2B—F)**. The models showed optimum agreement with true values across a wide concentration range, including samples with low and high levels of individual anthocyanins.

For total PHENs, the prediction model using the cross-validation method indicated a strong R^2^ value of 0.94 and a RPD value of about 4.3 **(**Fig. 5J-L**;** Table 2**)**. The spectral data after normalization using first derivative + multiplicative scattering correction (MSC) recorded a correlation in the region between 4597.8 and 9403.9 cm^−1^ with the total PHEN content. The model showed a slight overestimation in the predicted values, indicating a positive bias (0.6). The RMSECV value of 27.2 suggests a robust model, although with a slight variability in prediction accuracy. Notably, the model captured variations in average PHEN content ranged from 89.5 μg/100 g dw in samples with purple skin (:1000) to 270.3 μg/100 g dw in samples with purple root with non-purple phloem (:1101). Among the PHEN, the PLSR model for 3CQA and CA also demonstrated high predictive accuracy with R^2^ values of 0.93 each **(**Fig. 5K-L**;** Table 2**)**. These results are consistent with the study on NIR prediction of phenolic acids in coffee and beans (Carbas et al., 2020; Ribeiro et al., 2011). The application of the first derivative and SNV normalization improved spectral correlations across distinct wavelength regions. The 3CQA shows correlations at 6819.6–9403.9 cm^−1^ and 3610.4–4242.9 cm^−1^, while CA displayed association at 8887–9403.9 cm^−1^, 7336.4–7861 cm^−1^, and 4752.9–6827.3 cm^−1^. The estimates of total PHENs, 3CQA, and CA suggest strong concordance between the predicted and measured values with statistical significance at a 95 % confidence interval **(Table S6)**. Additionally, the quantified values of ACNs and PHEN contents using all the prediction models were able to distinguish all six purple binary phenotypes based on their predicted values **(Table S7)**. Overall, these findings suggest that NIRS, combined with optimized chemometric modelling, can serve as a rapid, non-destructive tool for screening anthocyanin and phenolic contents in a wide range of purple carrots.

### 3.4 Extraction efficiency of ACN and color stability

**Fig. 6:**
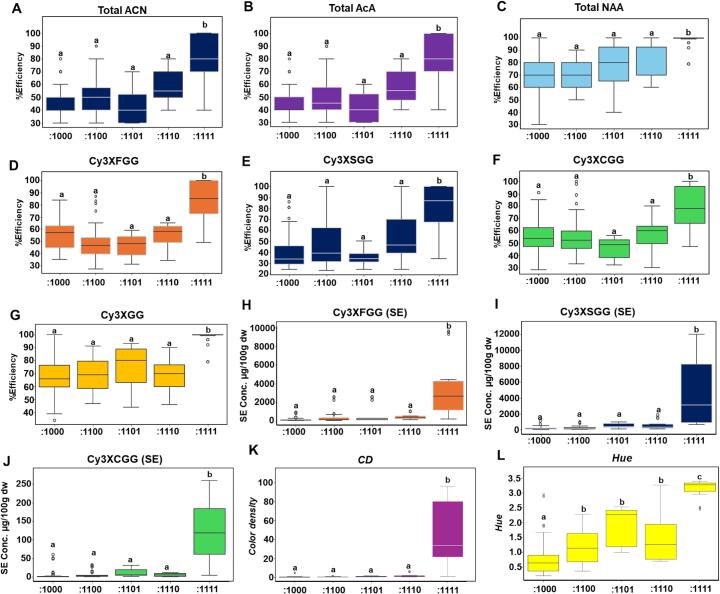
Extraction performance. Extraction efficiency of **(A)** total anthocyanins, **(B)** total acylated anthocyanins, **(C)** total non-acylated anthocyanins, **(D)** Cy3XFGG, **(E)** Cy3XSGG, **(F)** Cy3XCGG and **(G)** Cy3XGG across 5 binary purple phenotypes. **(H-J)** Concentration of acylated anthocyanins **(H)** Cy3XFGG, **(I)** Cy3XSGG and **(J)** Cy3XCGG after steeping extraction (SE) and across 5 binary purple phenotypes. **(K)** The color density and **(L)** hue across 5 binary phenotypes. Statistical significance was assessed using Tukey's HSD test, with different letters indicating significant differences at *p* < 0.01. (For interpretation of the references to color in this figure legend, the reader is referred to the web version of this article.)

The extraction efficiency and color stability of ACNs from purple carrot are critical for their application as natural colorants in the food color industry (Anandhi et al., 2024; Perez et al., 2024). The efficiency of ACN and PHEN extraction was evaluated in a set of 40 purple carrot accessions and commercial cultivars, categorized into five binary phenotypic groups (:1111,:1110,:1101,:1100 and:1000) using a steeping extraction method. The results indicated that total ACN, total AcA, and total NAA exhibited significantly higher extraction efficiency in fully purple roots (:1111) **(**Fig. 6A-C**)**. Extraction efficiency of total PHEN did not show significant differences across the five binary phenotypic groups in this set of samples. Among the ACNs, the highest extraction efficiency was recorded for the NAA, Cy3XGG (98 %) followed by AcAs, including Cy3XFGG (82.3 %), Cy3XSGG (79 %) and Cy3XCGG (78 %) in fully purple roots **(Table 3;** Fig. 6A-G**)**. Among AcAs, the maximum average content obtained after steeping extraction (SE) was for Cy3XSGG (4308 μg/100 g dw) followed by Cy3XFGG (3160.7 μg/100 g dw), whereas the lowest content was for Cy3XCGG (118.3 μg/100 g dw) **(**Fig. 6H-J**)**.

**Fig. 7:**
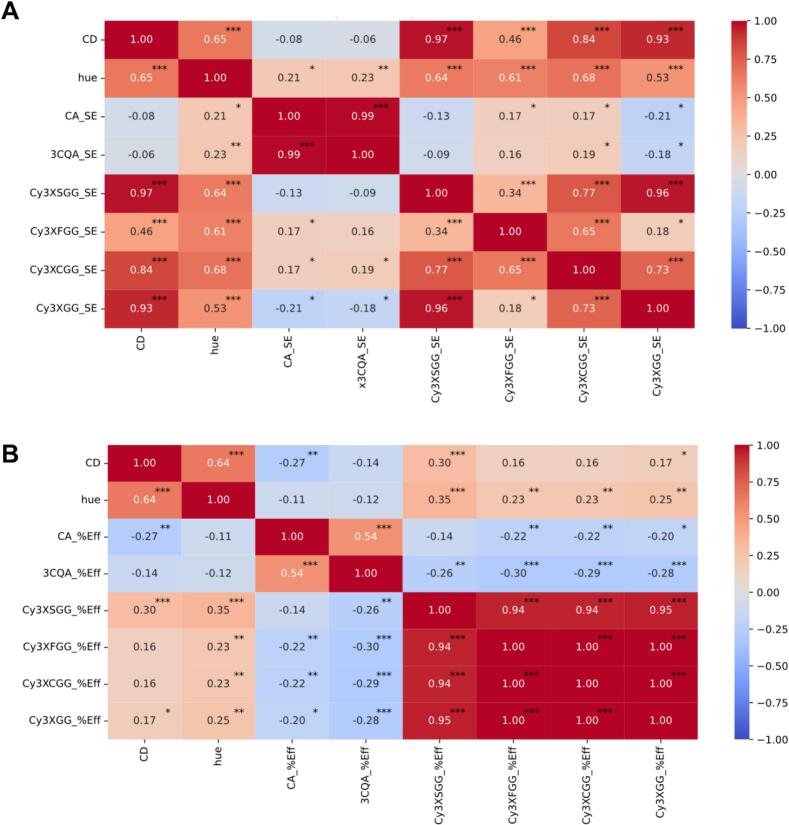
**(A)** Pearson's correlation between concentration of individual metabolites obtained after steeping extraction, color density and hues. **(B)** Pearson's correlation between extraction efficiency, color density and hues. The value in the plot and the heat map represent the Pearson's correlation coefficient (r), and significance is represented as *p < 0.05, **p < 0.01 and ***p < 0.001.

The pigment strength, quality and stability of extracts were measured using color density and hue, which are critical in food colorant industries. Color density (CD) measures the color intensity that reflects the combined contribution of both oxidized and non-oxidized ACNs, whereas hue (H), calculated as the ratio of absorbances, serves as a browning/oxidation index, providing insight into the structural and oxidative stability of ACN. The fully purple roots (:1111) recorded significantly higher value for both CD (avg. value: 43.5) and hue (3.2) as compared to all purple roots with one or more non-purple tissues **(**Fig. 6K-L**)**. The CD had a significantly strong correlation with ACNs, and no significant correlation with the PHEN (3CQA and CA) **(**Fig. 7A**)**. Among the AcA, Cy3XSGG exhibited the strongest positive correlation with CD (0.97), while Cy3XFGG had the lowest correlation (CD: 0.46). However, hue showed significantly higher correlations (*p* < 0.001) with the AcA Cy3XSGG (*r* = 0.64), Cy3XFGG (*r* = 0.61), and Cy3XCGG (*r* = 0.68) than with NAA and PHENs. While extraction efficiency for Cy3XSGG was lower compared to Cy3XGG, the higher absolute concentration and strong correlation with CD and hue suggest a key role in preserving visual attributes and antioxidant capacity by preventing browning/oxidation during steeping extraction of fully purple roots **(**Fig. 7A**)**. Additionally, the extraction efficiency of Cy3XSGG also demonstrated significantly higher correlations with CD (*r* = 0.30, p < 0.001) and hue (*r* = 0.35, p < 0.001) as compared to the other forms of AcA (Cy3XFGG, Cy3XCGG) and NAA (Cy3XGG) **(**Fig. 7B**)**. These findings add to earlier studies, where the increased methoxylation in the sinapoyl moiety of Cy3XSGG conferred greater pH and thermal stability of this compound compared to other acylated derivatives (Holme et al., 2021; Montilla et al., 2011). Although Cy3XFGG exhibited a low correlation with CD despite its higher extraction efficiency and absolute concentration, it had a significant correlation with hue. This suggests that Cy3XFGG may also play an important role in mitigating browning and anthocyanin oxidation.

Among all the samples tested in this experiment the fully purple variety ‘Black Nebula’ exhibited the highest extraction yield and moderately high efficiency of Cy3XSGG (79 %, 8367.4 μg/100 g dw) and Cy3XGG (100 %, 863.5 μg/100 g dw). Notably, the Cy3XSGG content was threefold higher than that of Cy3XFGG and accompanied by significantly elevated color density (CD: 81.9) and hue (H: 3.4). These findings suggest that ‘Black Nebula’ possesses a more intense and stable pigmentation, making it a valuable genetic resource for breeding programs aimed at developing Cy3XSGG-rich carrot cultivars (Duan et al., 2022). In contrast, the commercial variety ‘Deep Purple F1’ and ‘Moonrise Mix’ had 100 % extraction efficiency and a relatively higher extraction yield of Cy3XFGG (9480.3 μg/100 g dw and 4340.5 μg/100 g dw respectively) than Cy3XSGG (3333.3 μg/100 g dw and 2445.3 μg/100 g dw). Notably, these cultivars displayed low color density (‘Deep Purple F1’: 43.2 and ‘Moonrise Mix’: 31.0) and similar hue (‘Deep Purple F1’: 3.3 and ‘Moonrise Mix’: 3.1) suggesting that Cy3XSGG contributes significantly to increase CD (97 %) when in high amounts despite its lower relative abundance (%) compared to Cy3XFGG. In contrast, the accession ‘PI 204575’ with a lower ACN yield (Cy3XFGG: 2433 μg/100 g dw; Cy3XSGG: 904 μg/100 g dw) also had a substantially reduced CD (1.3) and hue (2.3). This further supports that Cy3XSGG content is a strong driver of CD.

## 4 Conclusion

Overall, the study indicated that selecting for carrots that have fully purple roots, with high amount of Cy3XSGG could increase ACN extraction yield, color density and ACN stability. Selecting for high PHENs as potential co-pigments to increase ACN stability is feasible, but it may require some extra steps in the selection process due to preferential accumulation of PHENs in root with non-purple phloem. Purple cultivars or accessions with great potential for producing high ACN yield and stability were identified. This material could be used as parents in breeding programs or tested in the field by growers for commercial purple carrot production. Finally, the study delivered prediction models for 10 key metabolites including total ACN, total AcA, Cy3XFGG, Cy3XSGG, Cy3XCGG, Cy3XG, Cy3XGG, total PHENs, 3CQA, and CA, enabling rapid non-destructive and cost-effective phenotyping for ACN. These outcomes offer valuable insight and tools for selecting ACN-rich accessions/cultivars suited for natural colorant extraction.

## CRediT authorship contribution statement

**Romit Seth:** Writing – original draft, Visualization, Software, Methodology, Investigation, Data curation. **Chelsey Fiecke:** Writing – review & editing, Methodology, Formal analysis. **Guoying Ma:** Writing – review & editing, Methodology. **Penelope Perkins-Veazie:** Writing – review & editing, Resources, Methodology. **Pablo Cavagnaro:** Writing – review & editing, Methodology. **Mario G. Ferruzzi:** Writing – review & editing, Supervision, Resources, Project administration, Investigation, Funding acquisition, Conceptualization. **Massimo Iorizzo:** Writing – review & editing, Supervision, Resources, Project administration, Investigation, Funding acquisition, Data curation, Conceptualization.

## Declaration of competing interest

The authors declare that they have no known competing financial interests or personal relationships that could have appeared to influence the work reported in this paper.

## Data Availability

No data was used for the research described in the article.
