# Supplementary material for: Characterization of anthocyanins, phenolics, and flavonoids in a global carrot collection through application of chemometrics and FT-NIR spectroscopy
Source: Food Chem X. 2025 Jul 18;29:102807. doi: 10.1016/j.fochx.2025.102807 (PMC12305724; doi:10.1016/j.fochx.2025.102807)
Supplement: Supplementary material 1 — Supplementary Figures S1-S2 [file mmc1.docx]

**Supplementary Figures**

**Characterization of anthocyanins, phenolics, and flavonoids in a global carrot collection through application of chemometrics and FT-NIR spectroscopy**

**Authors**

Romit Seth**^1^**, Chelsey Fiecke**^2^**, Guoying Ma**^1^**, Penelope Perkins-Veazie**^1,3^**, Pablo Cavagnaro**^4^**, Mario G. Ferruzzi**^2^** and Massimo Iorizzo**^1,3*^**

**Affiliations**

^1^ Plants for Human Health Institute, North Carolina State University, Kannapolis, North Carolina, United States

^2^Arkansas Children's Nutrition Center and Department of Pediatrics, University of Arkansas for Medical Sciences, Little Rock, AR, 72202, United States

^3^ Department of Horticultural Science, North Carolina State University, Raleigh, North Carolina, United States

^4^ National Scientific and Technical Research Council (CONICET), National Agricultural Technology Institute (INTA) E.E.A. Mendoza, Mendoza 5507, Argentina

(*) Corresponding Author: Massimo Iorizzo (miorizz@ncsu.edu)


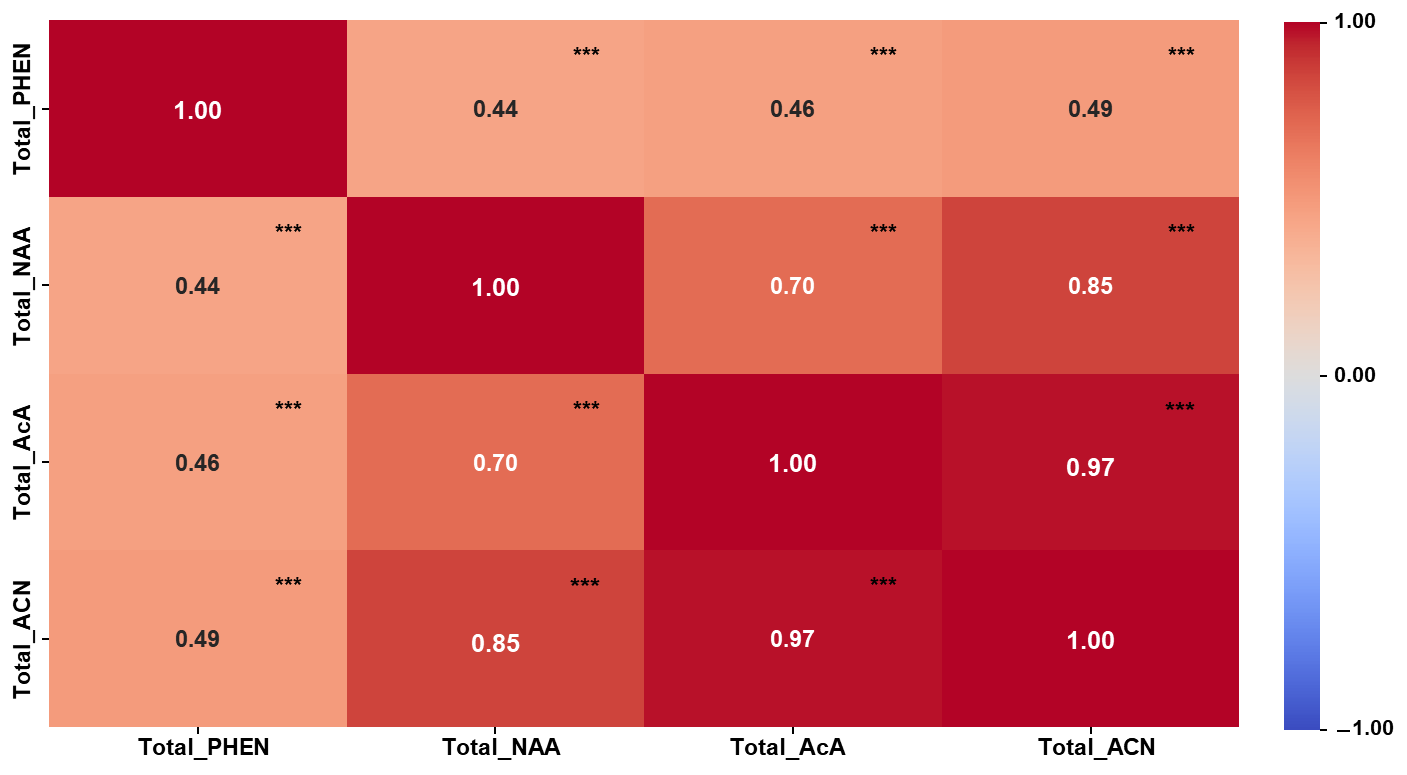


**Fig. S1:** Pearson’s correlation plot showing correlation among Total_ACN, Total_AcA, Total_NAA, and Total PHEN in set of purple carrot roots.The values in the plot represent the Pearson’s correlation coefficient (r), and significance is represented as *p<0.05, **p<0.01 and ***p<0.001.


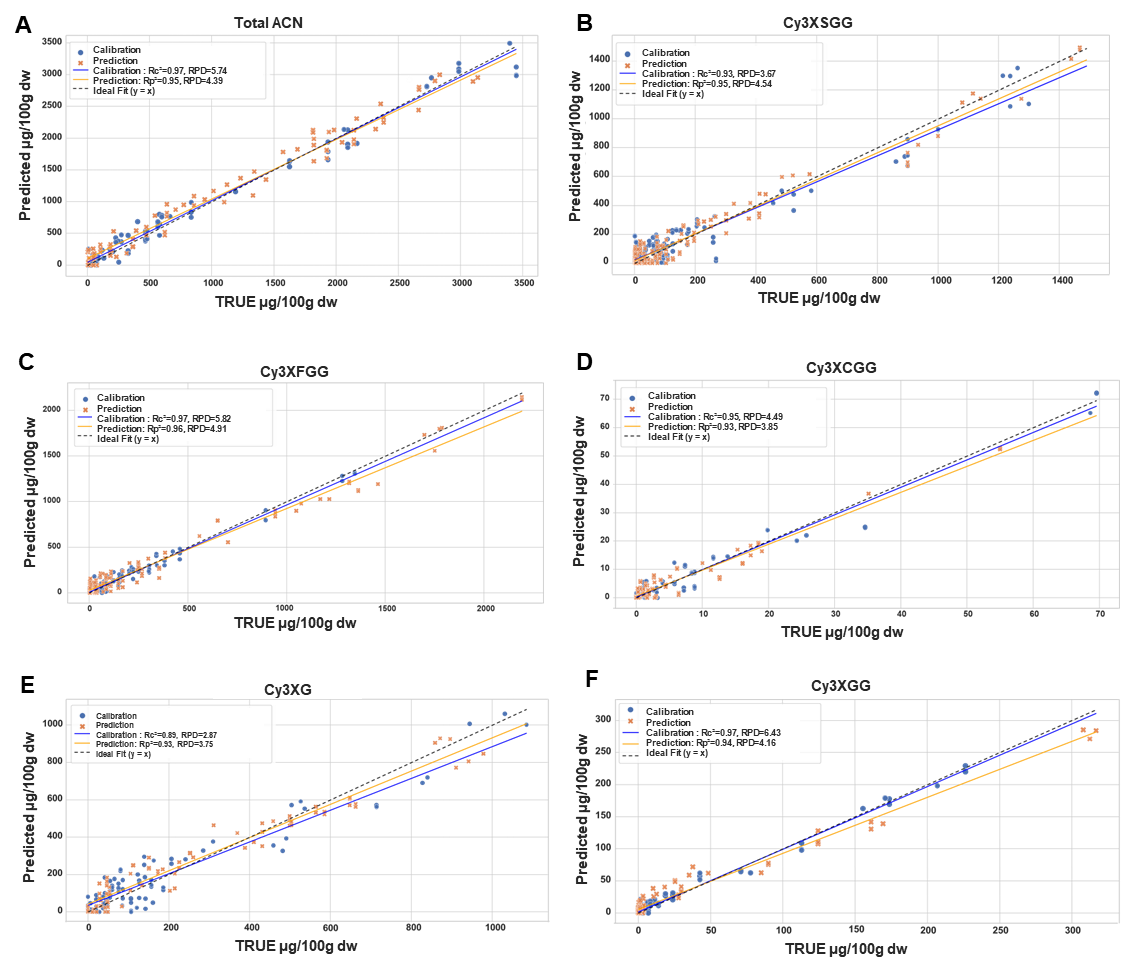


**Fig. S2:** Comparison of NIR calibration curve and validation of the predicted values for (A) Total ACN, (B) Cy3XSGG, (F) Cy3XFGG (G) Cy3XCGG; non acylated anthocyanins (H) Cy3XG & (I) Cy3XGG, using an external validation set. Rp^2^: Coefficient of determination in prediction; Rc^2^: Coefficient of determination in calibration; RPD: Residual prediction deviation from rank.
